# When the colon takes a hit: a rare case of acute colonic intramural hematoma after blunt abdominal trauma

**DOI:** 10.1093/jscr/rjag849

**Published:** 2026-09-22

**Authors:** Natasha L Frontera, Carlos Diaz Rivera, Fabiola A Ortega-Guzman, Agustin A Rodriguez Lopez, Luis A Santiago-Sulsona, Claudia B Sotomayor Rivera, Pablo Rodríguez-Ortiz

**Affiliations:** Department of Surgery, Menonita Health System, Carr 14, Barrio Rincon, Cayey 00736, Puerto Rico; Department of Surgery, University of Puerto Rico School of Medicine, Paseo Dr Jose Celso Barbosa, San Juan 00921, Puerto Rico; Department of Surgery, University of Puerto Rico School of Medicine, Paseo Dr Jose Celso Barbosa, San Juan 00921, Puerto Rico; Department of Surgery, University of Puerto Rico School of Medicine, Paseo Dr Jose Celso Barbosa, San Juan 00921, Puerto Rico; Department of Surgery, San Lucas Episcopal Health System, 917 Av Tito Castro, Ponce 00732, Puerto Rico; Department of Radiology, University of Puerto Rico School of Medicine, Paseo Dr Jose Celso Barbosa, San Juan 00921, Puerto Rico; Department of Surgery, University of Puerto Rico School of Medicine, Paseo Dr Jose Celso Barbosa, San Juan 00921, Puerto Rico

**Keywords:** colon bleeding, trauma, ascending colon

## Abstract

Acute colonic intramural hematomas are rare but clinically significant. They are associated with anticoagulation use, coagulopathies, and trauma. We report a case of a 20-year-old male who was referred to the trauma hospital from an outside institution with right lower quadrant pain and a palpable mass the day of a motor vehicle accident. Computed tomography and magnetic resonance imaging showed an intramural colonic hematoma (measuring 8.3 cm AP × 10.7 cm TR × 13.8 cm long). The patient was initially managed conservatively due to stable hemodynamic status and normal hemoglobin levels. However, the patient’s clinical status deteriorated and therefore underwent surgical intervention. Exploratory laparotomy confirmed a large intramural hematoma with a ruptured lateral cecal/ascending colonic wall. An open right hemicolectomy with ileocolonic anastomosis was performed. The final pathologic diagnosis was a hemorrhagic infarction of the colon. The patient recovered well and was discharged home 7 days after surgery.

## Introduction

Documentation of gastrointestinal trauma dates to the early 300s bc, when Aristotle utilized postmortem deer studies to describe the susceptibility of the intestinal wall to injury after blunt abdominal trauma (BAT) [1]. Intramural hematomas of the gastrointestinal tract in humans, however, were initially described in 1838 by McLaughlan [2]. These occur more commonly in the small bowel at the level of the duodenum but can also rarely occur in the large bowel. More specifically, acute colonic intramural hematomas (ACIH) have been reported, involving the cecum, ascending colon, sigmoid, and descending colon, secondary to the use of anticoagulation, coagulopathies (e.g. hemophilia, leukemia), or BAT [2–4]. We report a rare case of a 20-year-old male who presented with a post-traumatic acute colonic intramural hematoma of the cecum extending to the ascending colon and review the relevant literature so that increased awareness of the condition may guide future management standards.

## Case report

**Figure 1:**
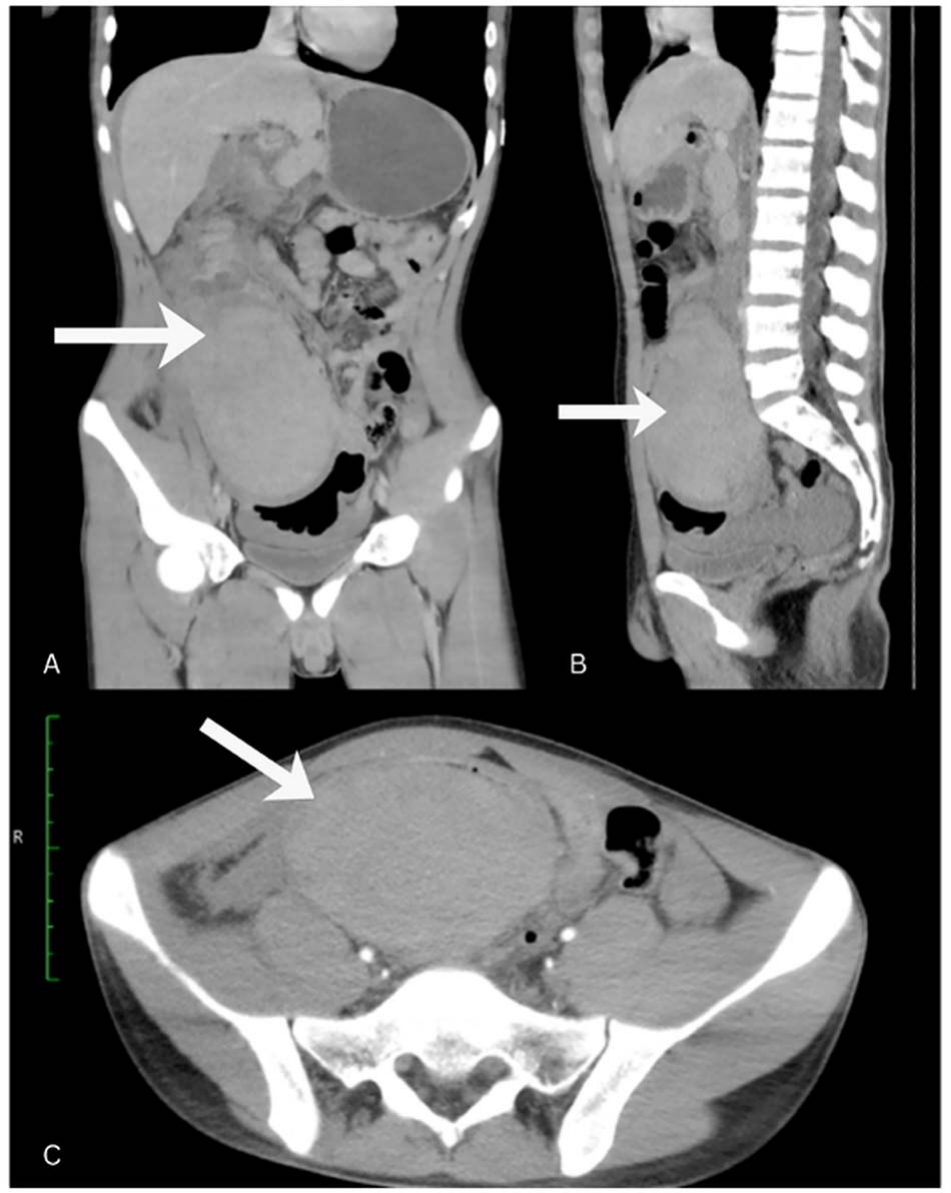
Contrast-enhanced computed tomography (CECT) of the abdomen showing an intramural hematoma (arrow) of the cecum and ascending colon measuring 7.9 cm AP × 9.8 cm TR ×14.4 cm long; (A) coronal view, (B) sagittal view, and (C) axial view.

A 20-year-old otherwise healthy male patient was transferred from an outside hospital to our trauma hospital at 10 a.m. after a motor vehicle accident 9 h prior. The patient reported nausea and vomiting at the referring hospital. Vital signs upon arrival were remarkable for tachycardia (heart rate = 115), but otherwise stable. Upon physical examination, the abdomen was depressible; no rebound or guarding was noted, with minimal right lower quadrant tenderness to palpation, and a palpable mass at the right iliac fossa. A focused assessment with sonography for trauma exam was positive for free fluid in the right upper abdominal quadrant. A computerized tomography (CT) scan from the outside hospital reported a left pneumothorax, an intraperitoneal hematoma without active bleeding in the right hemipelvis, and an L5 superior endplate fracture (Fig. 1). Laboratory results from the referring hospital revealed a hemoglobin level of 9.2 and a white blood cell count of 23 000.

**Figure 2:**
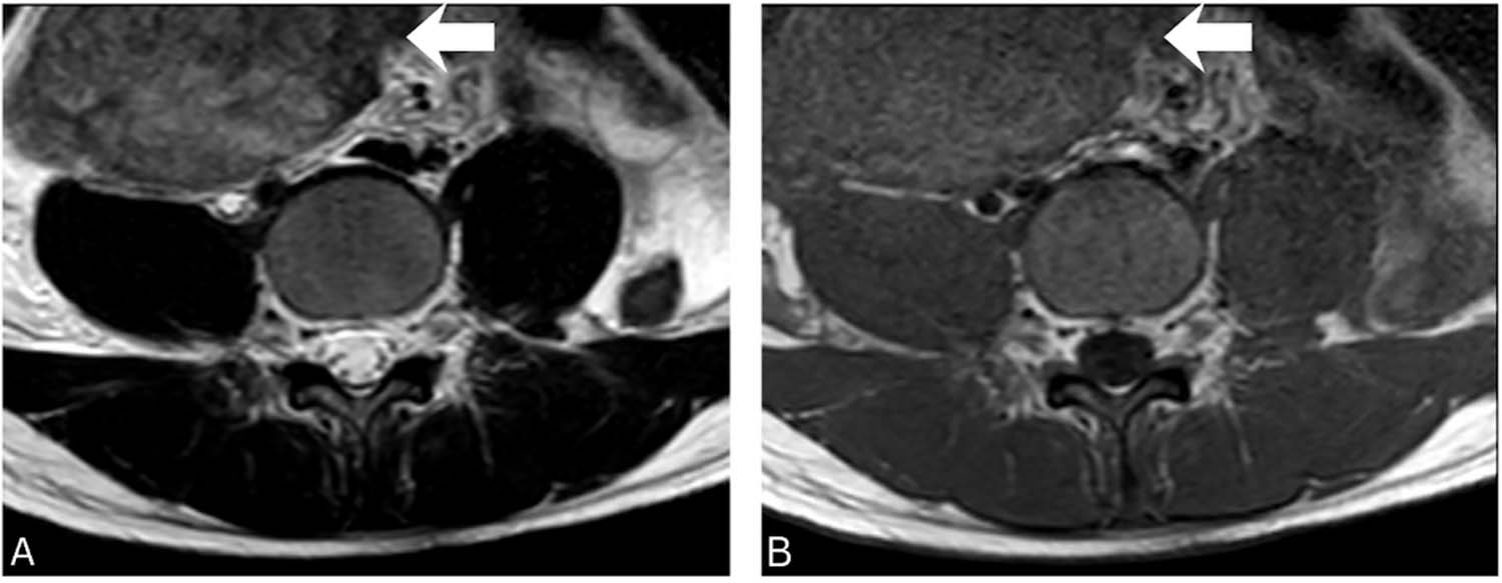
Axial view of a lumbar magnetic resonance image without contrast; (A) T2 weighted image shows heterogeneous intermediate signal intensity (arrow); (B) T1 weighted image (arrow).

**Figure 3:**
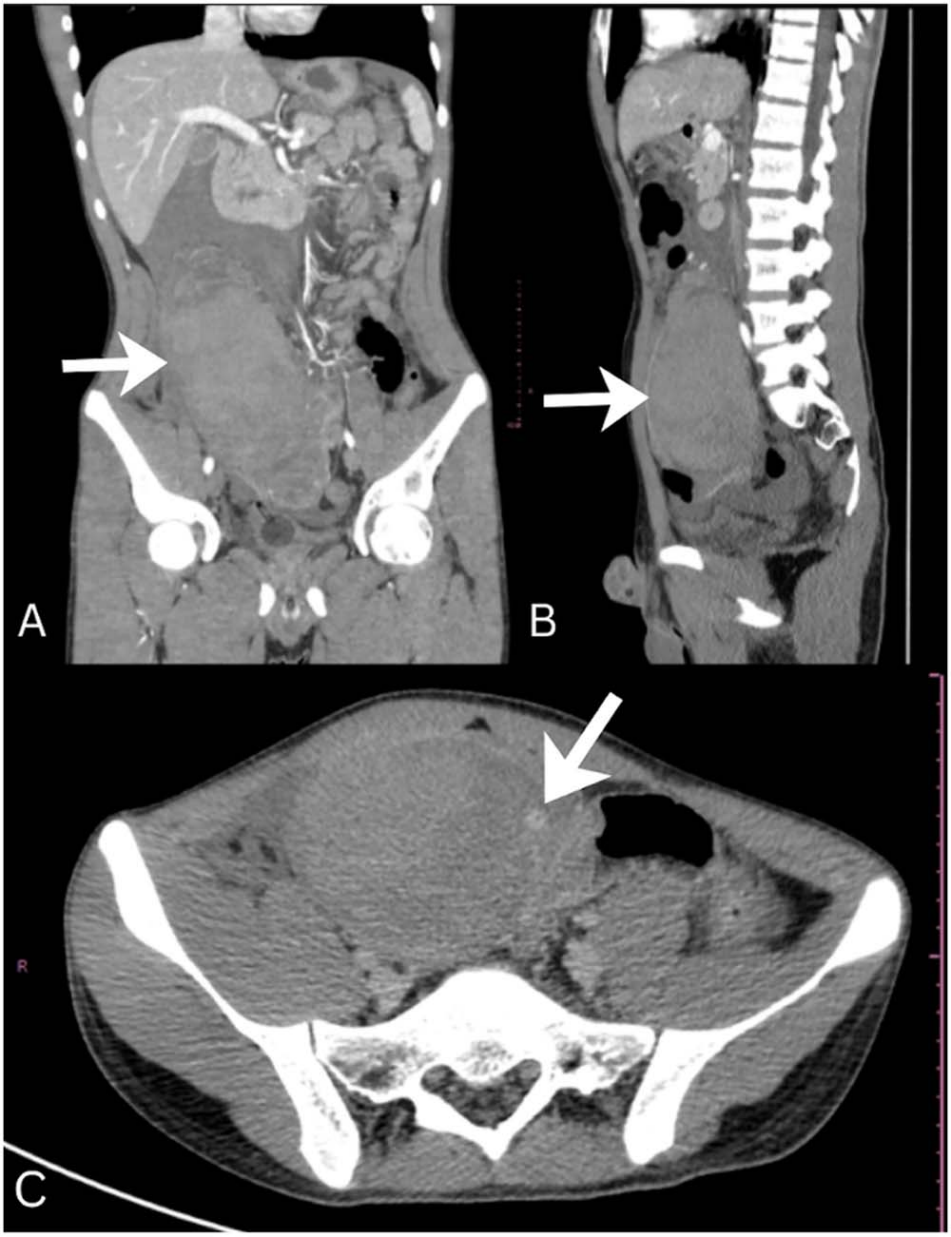
CECT of the abdomen showing an intramural hematoma (arrows) of the cecum and ascending colon measuring 8.3 cm AP × 10.7 cm TR × 13.8 cm long; (A) coronal view, (B) sagittal view, and (C) axial view show contrast pooling (arrow) of active extravasation consistent with active bleed.

Since the patient was hemodynamically stable and without signs of generalized peritonitis, he initially underwent non-operative management. Neurosurgery was consulted for the L5 fracture, and a lumbar magnetic resonance imaging without contrast was performed. The images revealed a Chance-type L5 fracture, as well as a large intramural hematoma involving the cecum and proximal ascending colon (Fig. 2). Due to increasing abdominal pain with episodes of emesis, an abdominopelvic CT scan with IV contrast was repeated at our institution ~12 h after arrival, which showed the cecal and ascending colon intramural hematoma with active bleeding and possible ischemic changes versus post-traumatic submucosal edema (Fig. 3).

Subsequently, ~30 h after admission, the patient’s hemoglobin began to decrease (from 9.2 to 6.7 g/dL), and the decision was made to transfuse two units of packed red blood cells and proceed with surgical intervention. Laparoscopic exploration was initially considered. However, because of the hemodynamic decline, the degree of hemoperitoneum on imaging, and the suspected extent of colonic injury, open exploratory laparotomy was deemed the safer and more appropriate approach.

**Figure 4:**
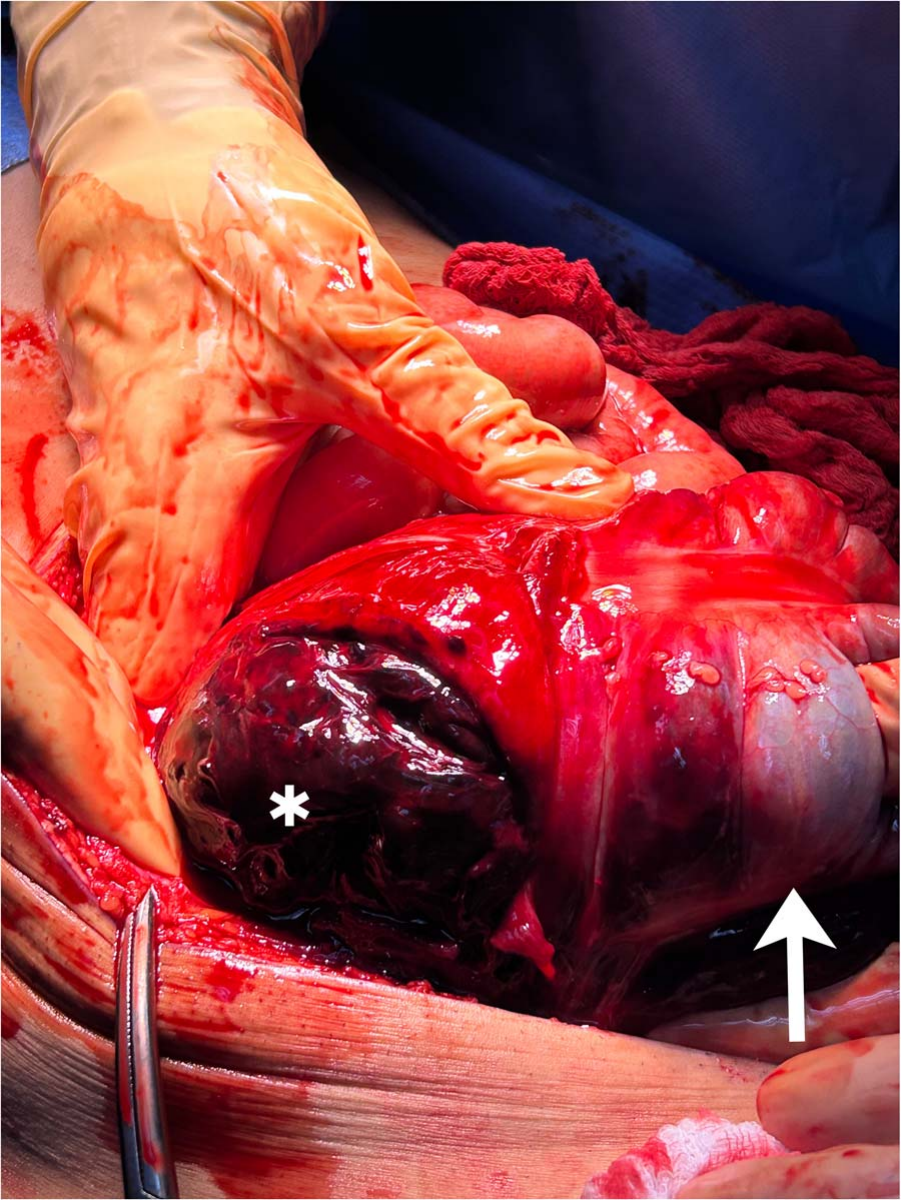
Intraoperative intramural hematoma (asterisk) and cecum (arrow).

**Figure 5:**
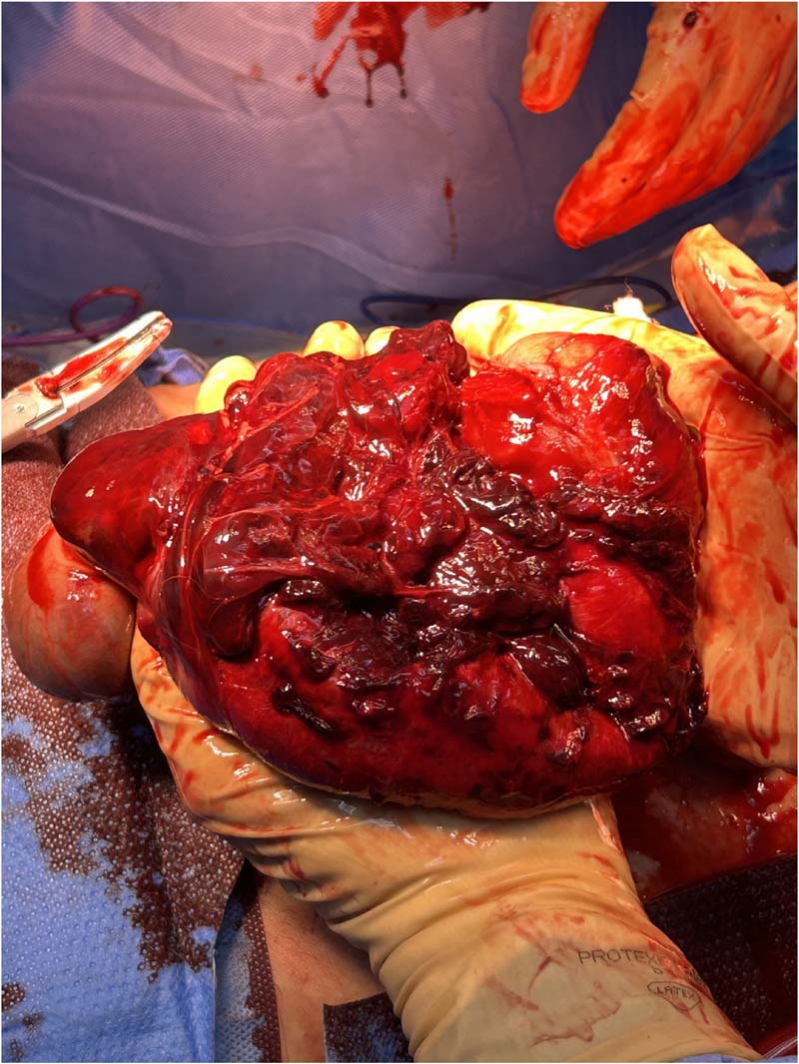
Evidence of the intramural hematoma as it ulcerated the colonic wall.

**Figure 6:**
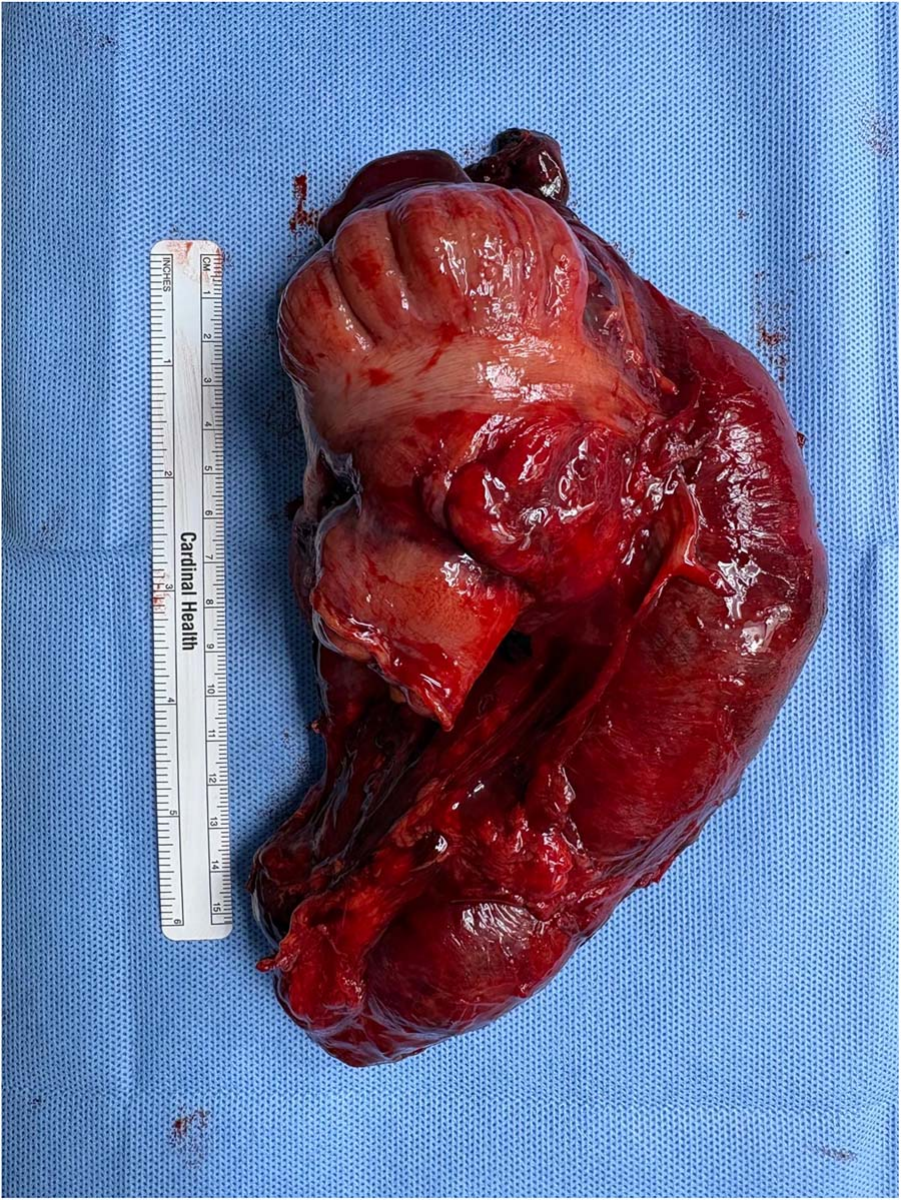
Ileocolonic specimen sent for pathological review.

Upon entry to the abdominal cavity, a moderate amount of hemoperitoneum was encountered. Intraoperative findings confirmed a severely dilated cecum with evidence of lateral ruptured colonic wall, with a large intramural hematoma extending by dissection of the tenia coli to the mid portion of the ascending colon (Figs 4–6). The surgery consisted of a right hemicolectomy with an ileocolonic anastomosis. The specimen was sent to pathology and a hemorrhagic infarction of the colon was confirmed. Postoperatively, intravenous fluids and antibiotics were administered. On post-operative Day 1, parenteral nutrition was started. The nasogastric tube was discontinued 2 days after surgery and enteral nutrition was started. The patient passed stool 5 days after surgery and was discharged home on post-operative Day 7.

## Discussion

**Figure 7:**
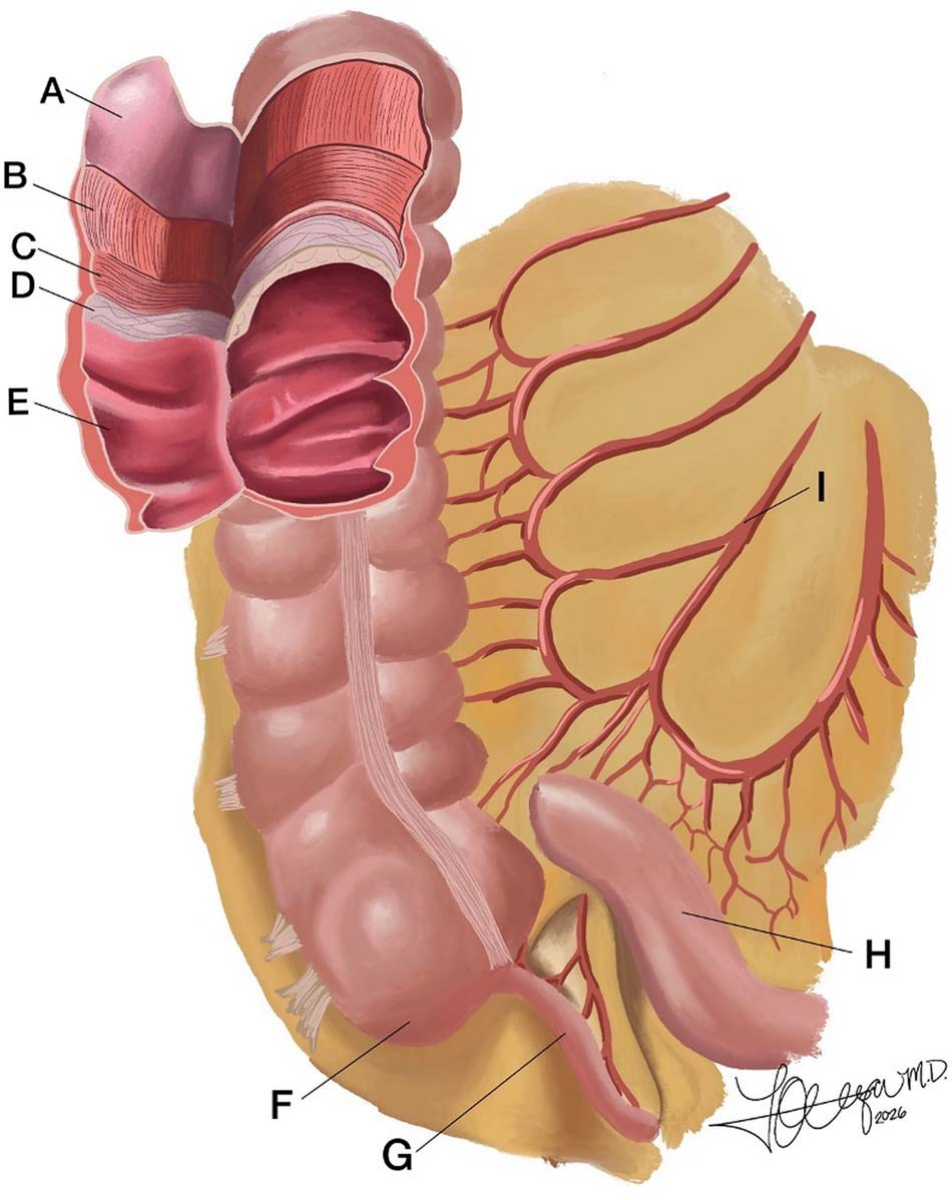
Anatomy of the colon: (A) serosa, (B) longitudinal muscle layer, (C) circular muscle layer, (D) submucosa, (E) mucosa, (F) cecum, (G) vermiform appendix, (H) terminal part of ileum, and (I) ileocolic artery (branch of superior mesenteric artery).

BAT induces shearing of the bowel wall layers due to decelerating or crushing forces in the submucosal vascular bed [2]. This leads to tearing of the terminal arterial vessels as they leave the mesentery and penetrate the muscularis layer (Fig. 7). Acute colonic intramural hematoma following BAT has rarely been reported. The low incidence may be attributed to the protective role of the tenia coli which can prevent blood diffusing in the bowel wall [2, 5].

The authors of this case report were able to identify only 13 cases of ACIH of the right colon published in the surgical literature since 1915 (Table 1) [2, 4–13]. Of the 13 cases, only one was a female patient. All patients were young (including three pediatric cases), and the age ranged from 8 to 37 years. The most frequent (*n* = 6) mechanism of injury was motor vehicle accidents. Other reported mechanisms included sports (*n* = 3), falls (*n* = 2), and blunt trauma with an object (*n* = 2). The most common anatomical location of the hematoma was the ascending colon (*n* = 6), followed by the combination of the ascending colon and cecum (*n* = 4), and the cecum only (*n* = 3). Regarding treatment, four of the cases were managed conservatively and the others underwent an operation; open colectomy, laparoscopic drainage, or open drainage and primary repair.

**Table 1:** Summary table of ACIH of right colon after BAT

**Author (ref)**	**Year**	**Sex**	**Age**	**Mechanism of injury**	**Location of hematoma**	**Treatment**
Bastionelli *et al*. (from reference [5])	1915	M	26	Bicycle handlebars	Cecum	Surgery
Nance and Crowder [6]	1968	M	29	MVA	Cecum	Surgery
Jeffrey *et al*. [7]	1982	M	33	MVA	Cecum/ascending colon	Surgery
Welling and Reilly [8]	1986	M	17	Football	Ascending colon	Conservative
Yin *et al*. [5]	1997	M	37	Stone	Cecum/ascending colon	Surgery
Calabuig *et al*. [9]	2002	M	21	Fall	Cecum/ascending colon	Surgery
Calabuig *et al*. [9]	2002	M	33	MVA	Cecum/ascending colon	Surgery
Hou and Tsou [10]	2009	M	27	MVA	Ascending colon	Conservative
Torres *et al*. [4]	2016	M	27	Soccer	Ascending colon	Surgery
Aaron *et al*. [2]	2020	M	28	MVA	Ascending colon	Conservative
Alzeerelhouseini *et al*. [11]	2021	M	8	Fall	Cecum	Surgery
Ibraheem *et al*. [12]	2024	F	14	MVA	Ascending colon	Conservative
Möller *et al*. [13]	2025	M	31	Kick to abdomen	Ascending colon	Surgery

Like other reported cases, our patient presented to the trauma hospital with the usual clinical presentation of an acute colonic intramural hematoma following BAT, including right lower quadrant pain, nausea, vomiting, right lower quadrant palpable mass, and leukocytosis. Of significance, the present case is only the fourth pediatric patient with ACIH after BAT reported in the literature. To our knowledge, it is also the only pediatric case treated successfully with a right hemicolectomy and primary ileocolonic anastomosis. As shown in Table 1, two of the other pediatric cases were treated conservatively, and one underwent primary evacuation and repair of the hematoma. Conservative treatment, although successful in four of the 13 reported cases, was not effective in our patient and, like most reported cases, he needed surgical intervention. If conservative treatment is implemented, the patient must be closely monitored for hemodynamic decline.

This is the first case of acute colonic intramural hematoma after BAT reported at our trauma hospital. Based on the available evidence and described cases in the literature, this condition remains rare and poorly understood. Our research hopes to create more awareness of the condition to help guide management effectively for optimal patient care.

## References

[1] Hughes CE 3rd, Conn J Jr, Sherman JO. Intramural hematoma of the gastrointestinal tract. Am J Surg 1977;133:276–9. 10.1016/0002-9610(77)90528-1 848656

[2] Aaron DJ, Bhattarai S, Shaikh O et al. Traumatic acute colonic intramural hematoma: a rare entity and successful expectant approach. Cureus 2020;12:e9694. 10.7759/cureus.9694 PMC7486096 32923284

[3] Westcott JL, Smith JRV. Mesentery and colon injuries secondary to blunt trauma. Radiology 1975;114:597–600. 10.1148/114.3.597 1118562

[4] Torres US, Cesar DN, D’Ippolito G. Computed tomography and magnetic resonance imaging findings in a case of colonic intramural hematoma after mild blunt abdominal trauma. J Comput Assist Tomogr 2016;40:896–8. 10.1097/RCT.0000000000000534 27759601

[5] Yin WY, Gueng MK, Huang SM et al. Acute colonic intramural hematoma due to blunt abdominal trauma. Int Surg 2000;85:51–4. 10817432

[6] Nance FC, Crowder VH. Intramural hematoma of the colon following blunt trauma to the abdomen. Am Surg 1968;34:85–7. 5635306

[7] Jeffrey RB, Federle MP, Stein SM et al. Intramural hematoma of the cecum following blunt trauma. J Comput Assist Tomogr 1982;6:404–5. 10.1097/00004728-198204000-00032 7076936

[8] Welling RE, Reilly PS. Nonoperative treatment of a traumatic intramural hematoma of the ascending colon. South Med J 1986;79:1309–10. 10.1097/00007611-198610000-00030 3489997

[9] Calabuig R, Ortiz C, Sueiras A et al. Intramural hematoma of the cecum: report of two cases. Dis Colon Rectum 2002;45:564–6. 10.1007/s10350-004-6240-y 12006943

[10] Hou MM, Tsou YK. Gastrointestinal: acute colonic intramural hematoma after blunt abdominal trauma. J Gastroenterol Hepatol 2009;24:494. 10.1111/j.1440-1746.2009.05832.x 19335786

[11] Alzeerelhouseini HIA, Abuzneid YS, Aljabarein OY. Delayed presentation of intramural cecal hematoma with challenges in the treatment. A case report and review of the literature. Int J Surg Case Rep 2021;82:105884. 10.1016/j.ijscr.2021.105884 PMC8086017 33887649

[12] Ibraheem HS, Hashem MS, Ebrahim SH et al. A rare case of traumatic colonic intramural hematoma in Saudi Arabia. Cureus 2024;16:e51461. 10.7759/cureus.51461 PMC10758202 38169609

[13] Möller KS, Georgiou E, Gurdayal Y. Colonic obstruction secondary to trauma induced intramural haematoma: a rare case report. S Afr J Surg 2025;63:178–80. 10.36303/SAJS.02270 41055035

